# Fevers

**Published:** 1905-12-09

**Authors:** 


### FEVERS.

(*Continued from page* 150.)

**Typhoid Fever.**—The question of diet in typhoid fever is one which has led to endless controversy. At present the majority of practitioners are certainly in favour of a liquid diet, whilst fever persists. But from time to time protesting voices are raised against this routine treatment of enteric patients, and doubts as to its wisdom are expressed in which probably many sufferers from the disease would concur. The subject has recently been reviewed in its historical aspects by J. B. Nichols[4] of Washington. In the old days before the discrimination of typhoid fever as a separate entity, "fevers" were classed as sthenic, inflammatory, or phlogistic, and asthenic. But even in the latter the symptoms were by many held to be deceptive and to be really due to over excitement. Corresponding to these two classes two schools of treatment were developed, the "antiphlogistic" or reducing, and the stimulating. Although each school had its ardent supporters, belief in the phlogistic theory more generally prevailed, and consequently bleeding, purging, emesis, starvation, and other reducing remedies were heroically pushed. Sydenham, the great English physician (1624-1689) followed this line of treatment vigorously. He bled copiously and repeatedly and for diet gave "water gruel, barley gruel, and the like," eked out with occasional spoonfuls of broth and beer! In the eighteenth century a reaction occurred, bleeding fell into disfavour and a more liberal diet of strictly vegetable origin consisting of soft fruits and farinaceous food was permitted, animal food of all kinds being strictly tabooed. Free drinking of water, plain, acidulated, or flavoured, to the amount of three or four quarts daily, was encouraged, a feature of treatment which has been revised by at least one distinguished modern physician. In the early part of last century the antiphlogistic regimen, with or without large quantities of water, was pursued vigorously until Graves' day when a return was again made to more liberal farinaceous diet. But although Graves jokingly requested that his epitaph might be "He fed fevers," his diet according to modern standards was not generous. After the separation of typhoid fever as a distinct disease by Gerhard in America and Jenner in England, farinaceous diet was continued until about the late 'sixties and 'seventies of last century, when the present liquid diet, consisting chiefly of milk, was gradually adopted and came into nearly universal use. Previous to that time, milk, regarded by Hippocrates as objectionable in fevers, had been either altogether forbidden or used very sparingly and highly diluted. It is thus seen that treatment with milk has been gradually evolved out of the old erroneous phlogistic theories of the pathology of fever, but although such a diet is far more supporting than that enforced by our fathers, the question remains whether enteric patients are insufficiently or suitably fed. All are agreed that the typhoid diet should be nutritious, readily digested, and calculated to lead to the minimum of irritating residue. The question at issue is whether milk really fulfils these conditions. Although ingested as a liquid it quickly becomes partly solid in the stomach, and as such is passed on into the intestine, and Nichols maintains that well divided solid food is as readily digested and that meat and eggs are provocative of less rather than more intestinal fermentation, to prevent which is one of the prime considerations in the treatment of typhoid, and leave less residue. Nichols does not dispute the comprehensiveness of milk, nor its value, as a diet, but seeks to show that advance in treatment of the disease has taken the form of an increase of diet, although patients still may go hungry and half-starved, and that a blind adherence to milk and milk only is empirical and traditional, whereas it would be more enlightened to try the effect on the patient's well-being of a more liberal and varied diet. C. Clements[5] used Sanatogen as an addition to milk and found that it appeared to assist convalescence and asthenia while leading to no evil results. It has been suggested[6] that the lessened susceptibility to typhoid enjoyed by the Japanese as compared with European and American soldiers —for to this as well as to their excellent sanitary arrangements must be ascribed their relative freedom from the disease in the Manchurian campaigns —may be due to their being a gramnivorous and rice-eating rather than a flesh-eating and alcohol drinking race. A case of lobar pneumonia has been described by Phillips and Spilsbury[7] in the course of enteric fever, in which no tubercle bacilli or pneumococci were found, but minute abscesses in the lung contained bacillus typhosus. An interesting case has been reported in Paris[8] in which symptoms of perforation occurred suddenly in an enteric patient in whom at the operation and also at the subsequent autopsy no sign of perforation could be anywhere detected, although signs of peritonitis, of older date than the origin of the symptoms, were well marked. The occurrence of peritonitis was ascribed to infection by contiguity from the intestine in this case, and not, as Dieulafoy explains such cases, as being due to an independent bacillary infection of the peritoneum such as may occur in the pleura or the bones.

[4] Med. Rec., July 29. [5] Lancet, July 1. [6] Ibid., May 20, p. 1366. [7] Brit. Med. Jour., May 20, p. 1093. [8] Lancet, May 20, p. 1368.

(*To be continued.*)

